# Assessment of 25-Year Survival of Women With Estrogen Receptor–Positive/*ERBB2*-Negative Breast Cancer Treated With and Without Tamoxifen Therapy: A Secondary Analysis of Data From the Stockholm Tamoxifen Randomized Clinical Trial

**DOI:** 10.1001/jamanetworkopen.2021.14904

**Published:** 2021-06-30

**Authors:** Huma Dar, Annelie Johansson, Anna Nordenskjöld, Adina Iftimi, Christina Yau, Gizeh Perez-Tenorio, Christopher Benz, Bo Nordenskjöld, Olle Stål, Laura J. Esserman, Tommy Fornander, Linda S. Lindström

**Affiliations:** 1 Department of Oncology and Pathology, Karolinska Institutet and University Hospital, Stockholm, Sweden; 2 Department of Oncology, Institute of Clinical Sciences, Sahlgrenska Academy, Gothenburg, Sweden; 3 Department of Medicine, Southern Älvsborg Hospital, Borås, Sweden; 4 Department of Biosciences and Nutrition, Karolinska Institutet, Stockholm, Sweden; 5 Department of Surgery, University of California, San Francisco, San Francisco; 6 Department of Biomedical and Clinical Sciences, Linköping University, Linköping, Sweden; 7 Department of Oncology, Linköping University, Linköping, Sweden; 8 Department of Medicine, University of California, San Francisco, San Francisco

## Abstract

**Importance:**

Clinically used breast cancer markers, such as tumor size, tumor grade, progesterone receptor (PR) status, and Ki-67 status, are known to be associated with short-term survival, but the association of these markers with long-term (25-year) survival is unclear.

**Objective:**

To assess the association of clinically used breast cancer markers with long-term survival and treatment benefit among postmenopausal women with lymph node–negative, estrogen receptor [ER]–positive and *ERBB2*-negative breast cancer who received tamoxifen therapy.

**Design, Setting, and Participants:**

This study was a secondary analysis of data from a subset of 565 women with ER-positive/*ERBB2*-negative breast cancer who participated in the Stockholm tamoxifen (STO-3) randomized clinical trial. The STO-3 clinical trial was conducted from 1976 to 1990 and comprised 1780 postmenopausal women with lymph node–negative breast cancer who were randomized to receive adjuvant tamoxifen therapy or no endocrine therapy. Complete 25-year follow-up data through December 31, 2016, were obtained from Swedish national registers. Immunohistochemical markers were reannotated in 2014. Data were analyzed from April to December 2020.

**Interventions:**

Patients in the original STO-3 clinical trial were randomized to receive 2 years of tamoxifen therapy vs no endocrine therapy. In 1983, patients who received tamoxifen therapy without cancer recurrence during the 2-year treatment and who consented to continued participation in the STO-3 study were further randomized to receive 3 additional years of tamoxifen therapy or no endocrine therapy.

**Main Outcomes and Measures:**

Distant recurrence–free interval (DRFI) by clinically used breast cancer markers was assessed using Kaplan-Meier and multivariable Cox proportional hazards analyses adjusted for age, period of primary diagnosis, tumor size (T1a and T1b [T1a/b], T1c, and T2), tumor grade (1-3), PR status (positive vs negative), Ki-67 status (low vs medium to high), and STO-3 clinical trial arm (tamoxifen treatment vs no adjuvant treatment). A recursive partitioning analysis was performed to evaluate which markers were able to best estimate long-term DRFI.

**Results:**

The study population comprised 565 postmenopausal women (mean [SD] age, 62.0 [5.3] years) with lymph node–negative, ER-positive/*ERBB2*-negative breast cancer. A statistically significant difference in long-term DRFI was observed by tumor size (88% for T1a/b vs 76% for T1c vs 63% for T2 tumors; log-rank *P* < .001) and tumor grade (81% for grade 1 vs 77% for grade 2 vs 65% for grade 3 tumors; log-rank *P* = .02) but not by PR status or Ki-67 status. Patients with smaller tumors (hazard ratio [HR], 0.31 [95% CI, 0.17-0.55] for T1a/b tumors and 0.58 [95% CI, 0.38-0.88] for T1c tumors) and grade 1 tumors (HR, 0.48; 95% CI, 0.24-0.95) experienced a significant reduction in the long-term risk of distant recurrence compared with patients with larger (T2) tumors and grade 3 tumors, respectively. A significant tamoxifen treatment benefit was observed among patients with larger tumors (HR, 0.53 [95% CI, 0.32-0.89] for T1c tumors and 0.34 [95% CI, 0.16-0.73] for T2 tumors), lower tumor grades (HR, 0.24 [95% CI, 0.07-0.82] for grade 1 tumors and 0.50 [95% CI, 0.31-0.80] for grade 2 tumors), and PR-positive status (HR, 0.38; 95% CI, 0.24-0.62). The recursive partitioning analysis revealed that tumor size was the most important characteristic associated with long-term survival, followed by clinical trial arm among patients with larger tumors.

**Conclusions and Relevance:**

This secondary analysis of data from the STO-3 clinical trial indicated that, among the selected subgroup of patients, tumor size followed by tumor grade were the markers most significantly associated with long-term survival. Furthermore, a significant long-term tamoxifen treatment benefit was observed among patients with larger tumors, lower tumor grades, and PR-positive tumors.

## Key Points

**Question:**

Are clinically used markers of breast cancer, such as tumor size, tumor grade, progesterone receptor status, and Ki-67 status, independently associated with 25-year survival and tamoxifen treatment benefit among patients with breast cancer?

**Findings:**

In this secondary analysis of data from 565 postmenopausal women with lymph node–negative, estrogen receptor–positive, and *ERBB2*-negative breast cancer who participated in the Stockholm tamoxifen randomized clinical trial (STO-3), tumor size and tumor grade were significantly associated with long-term (25-year) survival. A significant tamoxifen treatment benefit was observed among patients with larger tumors, lower tumor grades, and progesterone receptor–positive tumors.

**Meaning:**

This study’s findings suggest that tumor size and tumor grade is associated with long-term survival, and patients with larger tumors, lower tumor grades, and progesterone receptor–positive status experienced significant treatment benefit with receipt of tamoxifen therapy.

## Teaser

This secondary analysis of the Stockholm tamoxifen clinical trial examines the association of clinical breast cancer markers with 25-year survival and tamoxifen treatment benefit among postmenopausal women with lymph node–negative ER-positive/*ERBB2*-negative breast cancer.

## Introduction

Breast cancer is the most common female cancer in the Western world and one of the major causes of death among women. It is widely recognized as a heterogeneous disease with a long natural history.^1^ Over the past decades, a gradual increase in survival has been observed because of early detection, precise diagnosis, and improved treatment. Among women with estrogen receptor (ER)–positive tumors, treatment with adjuvant endocrine therapy is generally recommended to reduce the risk of recurrence and improve survival.^2^ However, approximately one-half of patients with ER-positive disease do not benefit from endocrine therapy, and approximately 1 in 4 patients later develop distant metastasis and die of breast cancer.^3,4^

Clinically used breast cancer markers are known to provide short-term survival estimates for up to 10 years after primary diagnosis. Numerous studies have reported that large tumors,^5^ high tumor grades,^6,7,8,9^ and high Ki-67 expression are associated with worse short-term survival.^10,11,12^ Tumor size and tumor grade are also routinely used to make decisions about adjuvant treatment, whereas the association between Ki-67 status and treatment benefit from endocrine therapy remains unclear.^13,14,15^ In addition, studies have indicated that progesterone receptor (PR) status might not provide independent information about prognosis in combination with other breast cancer markers,^16^ and the predictive value of PR has been debated.^2,17^

Patients with ER-positive tumors have a continuous long-term risk of distant recurrence and death compared with patients with ER-negative tumors.^3,4,18,19,20^ A study from the Early Breast Cancer Trialists’ Collaborative Group found that the risk of distant recurrence continues steadily throughout the 5 to 20 years after primary diagnosis.^4^ Among women with smaller tumors and lymph node–negative disease (ie, T1N0), a cumulative risk of 13% for distant recurrence was reported.^4^ The reasons for this long-term risk are unclear; however, it has been suggested that late fatal disease mechanisms may involve cancer cells remaining dormant over a long period.^21^ Given the late onset of fatal disease among those with ER-positive breast cancer, it is challenging to estimate patients’ long-term risk of fatal disease, and the ability of clinically used markers to independently estimate the long-term benefit of endocrine therapy has not been established.

Clinically used breast cancer markers are known to be associated with patient survival for up to 10 years after diagnosis.^16^ However, the association of these markers with long-term survival has not been established, and there are few well-annotated clinical studies with long-term follow-up data available. Because patients with ER-positive and *ERBB2* (formerly *HER2*; OMIM 164870)–negative (ER-positive/*ERBB2*-negative) disease have continuous risk for several decades after primary diagnosis,^4^ it is important to examine the long-term survival impact of primary breast cancer tumor characteristics, including clinically used markers of breast cancer. This study therefore aimed to assess whether clinically used markers were associated with long-term survival and tamoxifen treatment benefit among patients with lymph node–negative, ER-positive/*ERBB2*-negative breast cancer by performing a secondary analysis of data from the Stockholm tamoxifen (STO-3) randomized clinical trial. This large clinical trial provided complete long-term follow-up data from patients randomized to receive adjuvant tamoxifen therapy or no endocrine therapy.

## Methods

### The Stockholm Tamoxifen Clinical Trial

The Stockholm Breast Cancer Study Group has conducted randomized clinical trials since 1976.^22,23^ The STO-3 clinical trial enrolled 1780 postmenopausal women with lymph node–negative breast cancer and tumors with a diameter of 30 mm or less between 1976 and 1990. Patients were randomized to receive adjuvant tamoxifen therapy (40 mg daily) or no endocrine therapy (eMethods in Supplement 1). In 1983, patients who received tamoxifen therapy without cancer recurrence during the 2-year treatment and who consented to continued participation in the STO-3 study were further randomized to receive 3 additional years of tamoxifen therapy or no endocrine therapy. The STO-3 clinical trial, which was conducted at the Regional Cancer Center Stockholm-Gotland in Stockholm, Sweden, began in 1976, well before clinical trial registration started in Sweden; therefore, information on registration number was not available. The study was approved by the ethics committee of the Karolinska Institutet, and all participants provided oral informed consent. The STO-3 clinical trial followed the Consolidated Standards of Reporting Trials (CONSORT) reporting guideline for randomized clinical trials.

**Figure 1.**
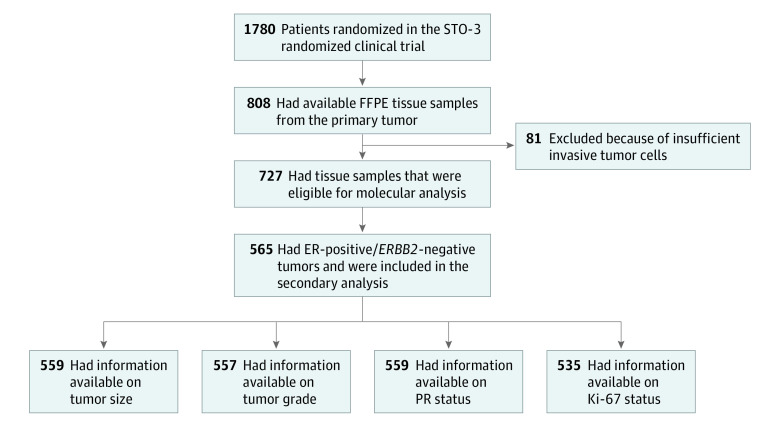
Participant Flowchart for Secondary Analysis of the Stockholm Tamoxifen (STO-3) Randomized Clinical Trial ER indicates estrogen receptor; FFPE, formalin-fixed, paraffin-embedded; PR, progesterone receptor.

Among the original 1780 patients, molecular analysis of tumors was possible for 808 patients who had formalin-fixed, paraffin-embedded tissue samples from the primary tumor available (Figure 1). The characteristics of this patient subset were well balanced with those of the original STO-3 clinical trial cohort (eg, 78% of patients in the subset vs 80% of patients in the original study cohort had ER-positive status) (eMethods in Supplement 1).^24^ Information on clinically used breast cancer markers (based on reannotation performed in 2014) was available for 727 patients in the STO-3 clinical trial; of those, 565 patients had a diagnosis of ER-positive/*ERBB2*-negative breast cancer and were included in this secondary analysis (Figure 1).

All residents in Sweden have a unique national registration number. This number allows automatic linkage with various personal records from national and regional registers, which provides high validity and essentially complete data coverage. Cancer registration is legally required in Sweden, and validation studies have reported that the Swedish Cancer Registry covers more than 96% of all cancer diagnoses in validation studies.^25^ Information on metastatic disease was obtained from the Regional Stockholm Breast Cancer Quality Registry of the Regional Cancer Center Stockholm-Gotland in Stockholm.^26^ Thus, through linkage with Swedish national and regional registers, complete long-term follow-up data from participants in the STO-3 clinical trial were available through December 31, 2016.

### Immunohistochemistry

Immunohistochemical analyses and reannotation of ER, PR, *ERBB2*, and antigen Ki-67 among participants in the STO-3 study were performed in 2014. Breast cancer pathologists scored the percentage of cancer cells with positive results for ER, PR, *ERBB2*, and Ki-67.^19^ A threshold of 10% or greater was used to define ER and PR positivity according to the Swedish *National Guidelines for Treatment of Breast Cancer*^27^; *ERBB2* positivity was defined as intensity of 3 or higher, and the Ki-67 threshold for medium to high expression was 15% or greater.

### Tumor Grade and Size

Tumor grade (1-3) was assessed in 2014 by 1 pathologist according to the Nottingham system (also known as Elston-Ellis grading).^24^ Tumor size was categorized into 3 groups based on clinical guidelines, with tumors of 10 mm or less classified as T1a and T1b (T1a/b), tumors of 11 mm to 20 mm classified as T1c, and tumors larger than 20 mm classified as T2.

### Statistical Analysis

#### Survival Analysis

An analysis of long-term distant recurrence–free interval (DRFI), as defined by Hudis et al,^28^ was performed by clinically used breast cancer markers, which comprised tumor size, tumor grade, PR status, and Ki-67 status. The outcome event was distant breast cancer recurrence. Patient follow-up started at the date of primary breast cancer diagnosis and ended at the date of distant breast cancer recurrence, death, emigration from Sweden (only 5 women emigrated), or December 31, 2016 (end of study follow-up), whichever occurred first. An analysis of long-term breast cancer–specific survival (BCSS) was also performed, with the outcome event defined as breast cancer–specific death (eTables 1 and 2 and eFigures 1-3 in Supplement 1).

Univariate Kaplan-Meier and multivariable Cox proportional hazards analyses of long-term (25-year) survival were also performed. Statistical significance for the Kaplan-Meier analysis was assessed using a log-rank test.^29^ The multivariable Cox proportional hazards model was adjusted for patient and tumor characteristics, which included STO-3 clinical trial arm and all available standard clinical markers known to be associated with breast cancer survival, such as age and period of primary breast cancer diagnosis, tumor size, tumor grade, PR status, and Ki-67 status.

#### Recursive Partitioning Analysis

A recursive partitioning analysis was performed to evaluate which of the clinically used breast cancer markers or patient characteristics were associated with long-term survival. A survival tree was constructed using the rpart package in R software, version 3.4.4 (R Foundation for Statistical Computing). Input variables in the model included age at primary breast cancer diagnosis, calendar period of primary breast cancer diagnosis, tumor size, tumor grade, PR status, Ki-67 status, and STO-3 clinical trial arm.

All data preparation and survival analyses were performed using SAS software, version 9.4 (SAS Institute Inc), and R software, version 3.4.4. Analyzed from April to December 2020.

## Results

The study population included 565 postmenopausal women (mean [SD] age, 62.0 [5.3] years) with a diagnosis of ER-positive/*ERBB2*-negative breast cancer (Figure 1). Of those, 520 patients (92.0%) had complete information available for all tumor characteristics (5 patients had missing information for >1 tumor characteristic) (eTable 1 in Supplement 1). With regard to tumor size, among 559 patients, 168 (30.0%) had T1a/b tumors, 292 (52.2%) had T1c tumors, and 99 (17.7%) had T2 tumors at primary diagnosis; of 557 patients, 128 (23.0%) had grade 1 tumors, 361 (64.8%) had grade 2 tumors, and 68 (12.2%) had grade 3 tumors (Table). Patient and tumor characteristics, including age and calendar period of primary breast cancer diagnosis, tumor size, tumor grade, PR status, and Ki-67 status, did not differ significantly between those who received tamoxifen therapy and those who did not (eTable 1 in Supplement 1).

**Table.** Risk of Distant Recurrence by Clinically Used Breast Cancer Markers^a^

Breast cancer marker	Total patients, No. (%)	Patients with distant recurrence over 25 y, No.	Risk of distant recurrence, HR (95% CI)
Tumor size (n = 559)			
T1a and T1b	168 (30.0)	20	0.31 (0.17-0.55)
T1c	292 (52.2)	63	0.58 (0.38-0.88)
T2	99 (17.7)	34	1 [Reference]
Tumor grade (n = 557)			
1	128 (23.0)	18	0.48 (0.24-0.95)
2	361 (64.8)	76	0.69 (0.41-1.15)
3	68 (12.2)	21	1 [Reference]
Progesterone receptor status^b^ (n = 559)			
Positive	391 (69.9)	77	0.85 (0.57-1.26)
Negative	168 (30.0)	39	1 [Reference]
Ki-67 status^c^ (n = 535)			
Low	427 (79.8)	86	0.85 (0.54-1.36)
Medium to high	108 (20.2)	28	1 [Reference]

Abbreviation: HR, hazard ratio.

^a^ Multivariable Cox proportional hazards model adjusted for age at primary diagnosis, calendar period of diagnosis, tumor size, tumor grade, progesterone receptor status, Ki-67 status, and STO-3 clinical trial arm.

^b^ Positive status was defined as progesterone receptor expression of 10% or greater.

^c^ The threshold for medium to high Ki-67 expression was 15% or greater.

### Univariate Analysis of Long-term Survival

**Figure 2.**
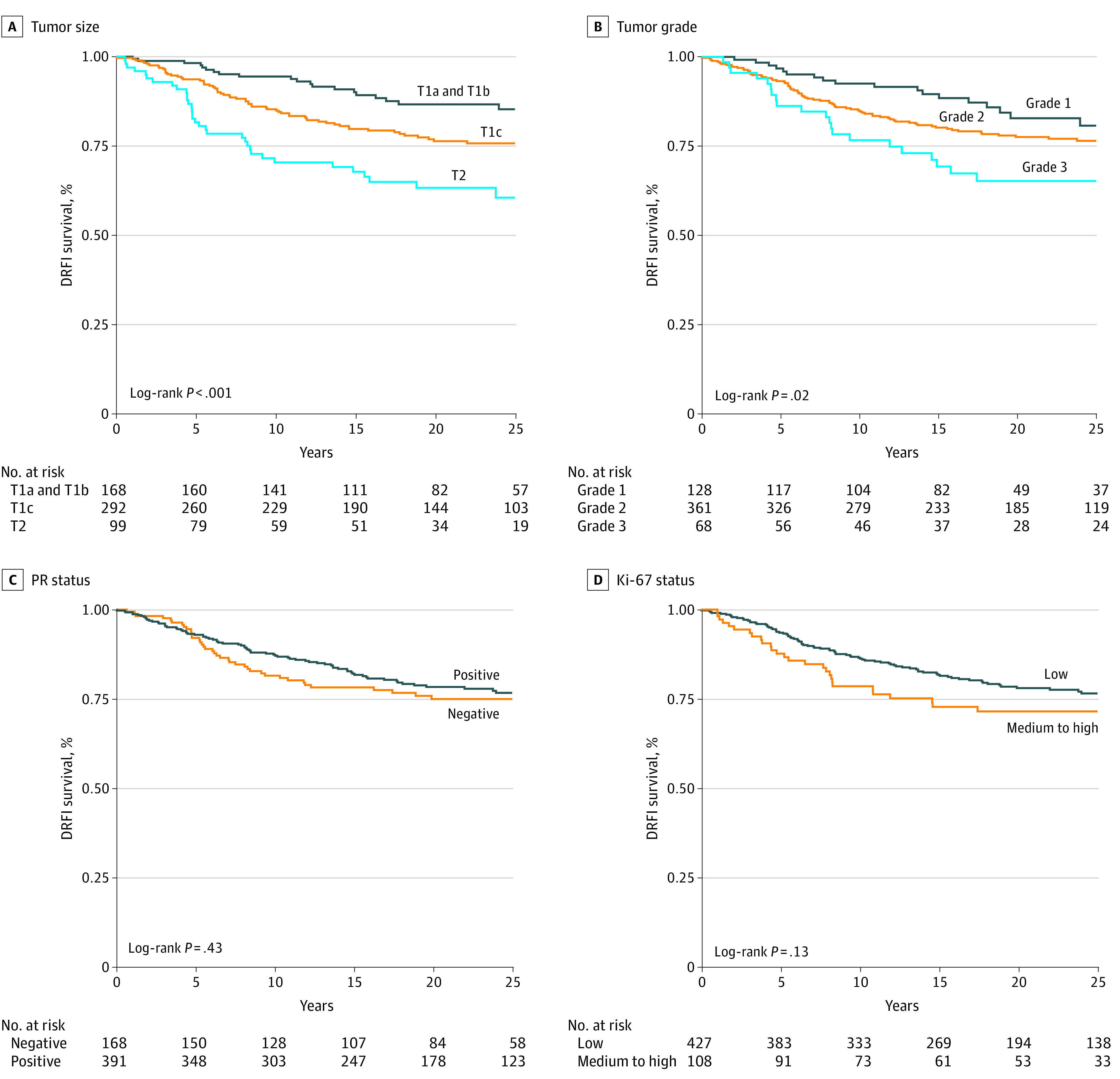
Kaplan-Meier Survival Analysis of Distant Recurrence–Free Interval (DRFI) The reported *P* values are based on a 2-sided log-rank test. PR indicates progesterone receptor.

A Kaplan-Meier analysis was performed by tumor size, tumor grade, PR status, and Ki-67 status. A statistically significant difference in long-term DRFI by tumor size was observed (Figure 2A). Patients with T1a/b tumors had the best long-term DRFI at 88% (95% CI, 80%-93%) compared with 76% (95% CI, 70%-81%) and 63% (95% CI, 50%-73%; log-rank *P* < .001) for patients with T1c and T2 tumors, respectively. A statistically significant difference in long-term DRFI by tumor grade was also found (Figure 2B). Patients with grade 1 tumors had the best long-term DRFI at 81% (95% CI, 70%-88%), followed by patients with grade 2 tumors at 77% (95% CI, 71%-81%). Patients with grade 3 tumors had the worst long-term DRFI at 65% (95% CI, 52%-76%; log-rank *P* = .02). A statistically significant difference in long-term DRFI by PR status and Ki-67 status was not found (Figure 2C and D). Similar results were observed for BCSS (eFigure 1 in Supplement 1).

### Multivariable Analysis of Long-term Survival

A multivariable Cox proportional hazards survival analysis for clinically used markers was performed to estimate long-term survival, adjusting for standard clinical patient and tumor characteristics. Consistent with the Kaplan-Meier analysis, a statistically significant reduction in the long-term risk of distant recurrence was found among patients with smaller tumors (T1a/b and T1c) vs larger tumors (T2) (hazard ratio [HR], 0.31 [95% CI, 0.17-0.55] for T1a/b tumors and 0.58 [95% CI, 0.38-0.88] for T1c tumors) (Table). Patients with grade 1 tumors had a reduced long-term risk of distant recurrence compared with patients with grade 3 tumors (HR, 0.48; 95% CI, 0.24-0.95). A statistically significant difference in long-term DRFI was not observed among patients with grade 2 vs grade 3 tumors (Table). Consistent with the Kaplan-Meier analyses, a statistically significant difference in long-term DRFI by PR status and Ki-67 status was not found (Table). Similar results were observed for BCSS (eTable 2 in Supplement 1).

### Multivariable Analysis of Long-term Treatment Benefit

**Figure 3.**
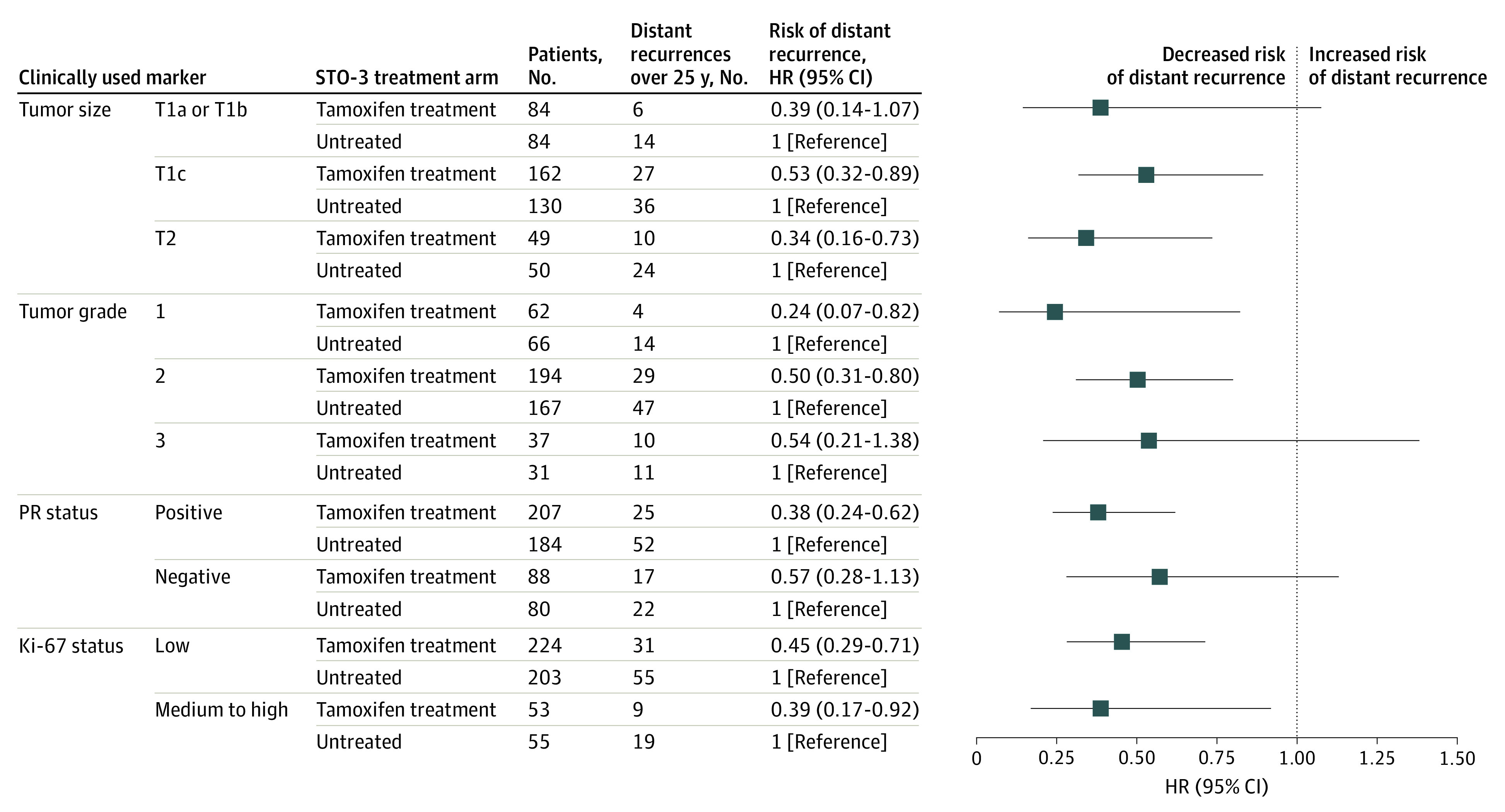
Distant Recurrence–Free Interval by Treatment Arm Forest plot showing risk of long-term (25-year) distant recurrence by Stockholm tamoxifen (STO-3) randomized clinical trial arm. Estimates were adjusted for patient and tumor characteristics. The horizontal lines indicate 95% CIs. HR indicates hazard ratio; PR, progesterone receptor.

A multivariable Cox proportional hazards survival analysis of clinically used markers by STO-3 clinical trial arm, adjusted for standard patient and tumor characteristics, was performed to estimate the long-term treatment benefit of tamoxifen therapy. A statistically significant reduction in long-term risk of distant recurrence was observed among patients with larger tumors who received tamoxifen treatment (HR, 0.53 [95% CI, 0.32-0.89] for T1c tumors and 0.34 [95% CI, 0.16-0.73] for T2 tumors) but not among those who had the smallest tumors (T1a/b) compared with patients who did not receive adjuvant treatment (Figure 3). Patients with grade 1 or grade 2 tumors who received tamoxifen therapy experienced a significant reduction in long-term risk of distant recurrence compared with those who did not receive adjuvant treatment (HR, 0.24 [95% CI, 0.07-0.82] for grade 1 tumors and 0.50 [95% CI, 0.31-0.80] for grade 2 tumors) (Figure 3). No significant treatment benefit was observed among patients with grade 3 tumors.

Patients with PR-positive disease who received tamoxifen treatment also experienced a reduction in the long-term risk of distant recurrence compared with patients who did not receive adjuvant treatment (HR, 0.38; 95% CI, 0.24-0.62). In contrast, patients with PR-negative disease had no significant long-term treatment benefit (Figure 3). Patients in the tamoxifen treatment arm who had medium to high Ki-67 expression (HR, 0.39; 95% CI, 0.17-0.92) and low Ki-67 expression (HR, 0.45; 95% CI, 0.29-0.71) had a reduced long-term risk compared with patients in the untreated arm (Figure 3). Similar results were observed for BCSS (eFigure 2 in Supplement 1).

### Recursive Partitioning Analysis

**Figure 4.**
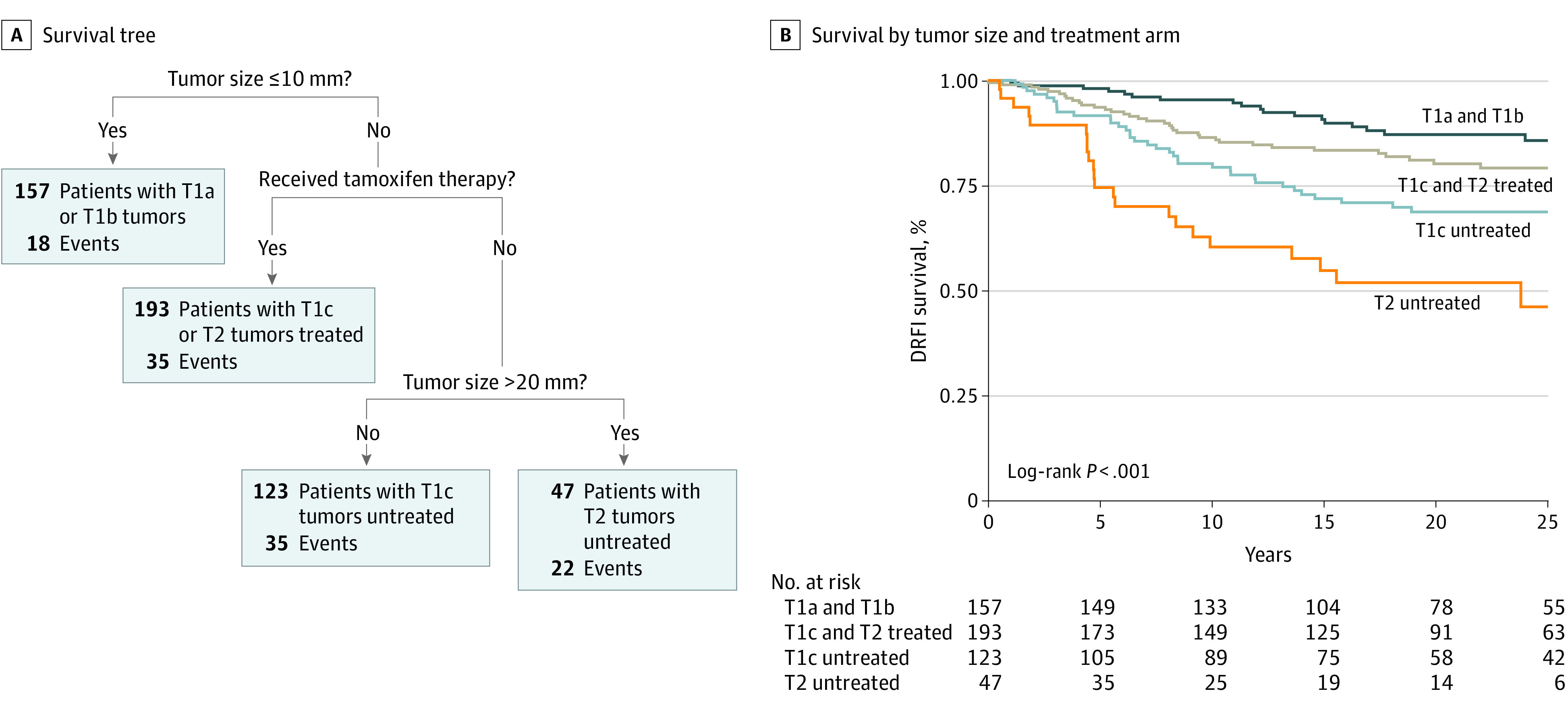
Recursive Partitioning Survival Tree and Kaplan-Meier Survival Analysis of Distant Recurrence–Free Interval (DFRI) The reported *P* value is based on a 2-sided log-rank test.

The recursive partitioning analysis first divided patients by tumor size, separating those with the smallest tumors (T1a/b) from those with larger tumors (T1c and T2) (Figure 4). Second, patients with larger tumors (T1c and T2) were further divided by clinical trial arm (tamoxifen treatment vs no adjuvant treatment). Third, patients with larger tumors who did not receive adjuvant treatment were further divided into tumor sizes T1c and T2. The final survival tree was selected by minimizing the cross-validation error. Patients with missing information on the selected breast cancer markers were excluded from the recursive partitioning model.

A statistically significant difference in long-term DRFI was observed in the Kaplan-Meier analysis of the recursive partitioning survival tree (86% for T1a/b tumors vs 79% for T1c and T2 treated tumors vs 69% for T1c untreated tumors vs 46% for T2 untreated tumors; log-rank *P* < .001) (Figure 4). The first division, which comprised patients with the smallest tumors (T1a/b), had the best long-term survival, followed by the second division, which comprised patients with larger tumors (T1c and T2) from the tamoxifen treatment group. The third division, which comprised patients who did not receive adjuvant treatment and who were separated by tumor size (T1c and T2), had the worst survival. Similar results were observed in the analysis of long-term BCSS (eFigure 3 in Supplement 1).

## Discussion

This secondary analysis of data from the STO-3 randomized clinical trial, investigated whether clinically used breast cancer markers were independently associated with long-term survival and tamoxifen treatment benefit. The findings indicated that, among this selected subgroup, tumor size and tumor grade were associated with long-term survival, and a significant tamoxifen treatment benefit was observed among patients with larger tumors, lower tumor grades, and PR-positive tumors. The STO-3 clinical trial follow-up data now enable DRFI outcome assessment for up to 25 years after primary diagnosis as well as examination of outcomes among those who received adjuvant tamoxifen therapy.

The findings of the present analysis suggest that tumor size is associated with the long-term risk of distant recurrence independent from other clinically used markers among patients with lymph node–negative, ER-positive/*ERBB2*-negative breast cancer. These findings are consistent with results from Pan et al,^4^ which included approximately 63 000 patients from different clinical trials with a 20-year follow-up and concluded that the risk of distant recurrence was associated with tumor size. Furthermore, the results of the present study suggest no long-term independent association with Ki-67 status among patients with lymph node–negative, ER-positive/*ERBB2*-negative breast cancer, and Ki-67 status was found to have only moderate estimation value in Pan et al^4^ (data on Ki-67 status were available for 12% of patients). The findings of the current study also suggest that tumor grade is associated with the long-term risk of distant recurrence, with a risk reduction observed among patients with grade 1 vs grade 3 tumors. In Pan et al,^4^ tumor grade was reported to have moderate estimation value for long-term distant recurrence. The Pan et al^4^ study had the advantage of including a large patient population, whereas the present study has the advantage of clinically used markers that were reannotated simultaneously and patients who received homogeneous treatment according to the STO-3 clinical trial arm.

The findings of this secondary analysis suggest that patients with larger tumors (T1c and T2) and lower tumor grades (1 and 2) receive substantial treatment benefit with tamoxifen therapy. Furthermore, a long-term treatment benefit was observed among patients with PR-positive tumors and in patients who had low and medium to high Ki-67 expression, which is consistent with the results of a previous STO-3 study.^17^ However, Davies et al^2^ found that the benefit of tamoxifen therapy among patients with ER-positive breast cancer was independent of PR status. There are several possible reasons for this difference in findings; the present study analyzed data from a larger single clinical trial that had limitations owing to the small number of patients available for subgroup analysis. It is also possible that the difference in findings is associated with variability in the determination of PR status in Davies et al^2^ owing to the variety of laboratories and techniques used to assess PR status. The difference in findings may also have occurred for other reasons, such as differences in patient populations (eg, the inclusion of only patients with lymph node–negative, ER-positive/*ERBB2*-negative breast cancer in the present analysis) or differences by treatment.^2,16^

To assess which of the clinically used markers was best able to estimate long-term survival, this study performed a recursive partitioning analysis to create a survival tree. The recursive model first selected tumor size as the most important characteristic associated with survival, and survival among patients with the smallest tumors (T1a/b) was solely estimated by tumor size. Among patients with larger tumors, treatment with tamoxifen therapy was the second most important variable associated with survival. Notably, findings from the resulting recursive partitioning model were consistent with the results from the Kaplan-Meier and multivariable analyses, indicating that tumor size is an important marker to understand long-term survival and tamoxifen treatment benefit.

### Limitations

This study has limitations. As with most long-term follow-up studies, clinical recommendations for disease management and treatment have changed since the initiation of the original clinical trial. The STO-3 clinical trial was performed before aromatase inhibitors became one of the recommended treatment options for patients with ER-positive breast cancer. In addition, when the STO-3 clinical trial was conducted, the duration of tamoxifen treatment was shorter, and the treatment dosage was higher than current recommendations. In the population-based STO-3 clinical trial cohort, approximately one-half of the patients had tumor samples available for molecular analysis. The present study therefore has limitations regarding the small number of patients available for subgroup analysis. We have, however, confirmed that patient and tumor characteristics in this secondary analysis were equally distributed and well balanced with those of the original STO-3 clinical trial cohort with regard to characteristics such as tumor size and ER status.^24^ In addition, when performing immunohistochemical analysis, there is often some level of inaccuracy. However, in the present study, the clinically used markers were stained at a single medical laboratory in 2014 and assessed by experienced breast cancer pathologists who had been harmonized with regard to the scoring of immunohistochemical markers.^30^

## Conclusions

The findings of this study indicate that, among patients with lymph node–negative, ER-positive/*ERBB2*-negative breast cancer from the STO-3 randomized clinical trial, tumor size followed by tumor grade were significantly associated with long-term risk of distant recurrence, as patients with larger tumors and higher tumor grades had significantly worse long-term survival compared to patients with smaller tumors and lower tumor grades. In contrast, PR status and Ki-67 status were not significantly associated with long-term survival in patients with lymph node-negative, ER-positive/ERBB2-negative breast cancer. The findings further indicated that, among this selected subgroup, a significant tamoxifen treatment benefit was observed among patients who had larger tumors, lower tumor grades, and PR-positive tumors.

## Notes

Supplement 1. **eMethods.** Stockholm Tamoxifen (STO-3) Clinical Trial and Estrogen Receptor, Progesterone Receptor, *ERBB2*, and Ki-67 Immunohistochemistry **eTable 1.** Patient and Breast Cancer Tumor Characteristics by Treatment Arm **eTable 2.** Breast Cancer–Specific Survival by Clinically Used Breast Cancer Markers **eFigure 1.** Kaplan-Meier Analysis of Breast Cancer–Specific Survival **eFigure 2.** Breast Cancer–Specific Survival by Treatment Arm **eFigure 3.** Recursive Partitioning Survival Tree of Breast Cancer–Specific Survival

Supplement 2. Data Sharing Statement

## References

[1] Pichon MF, Broet P, Magdelenat H, . Prognostic value of steroid receptors after long-term follow-up of 2257 operable breast cancers. Br J Cancer. 1996;73(12):1545-1551. doi:10.1038/bjc.1996.291 8664127 PMC2074541

[2] Davies C, Godwin J, Gray R, ; Early Breast Cancer Trialists’ Collaborative Group (EBCTCG). Relevance of breast cancer hormone receptors and other factors to the efficacy of adjuvant tamoxifen: patient-level meta-analysis of randomised trials. Lancet. 2011;378(9793):771-784. doi:10.1016/S0140-6736(11)60993-8 21802721 PMC3163848

[3] Colleoni M, Sun Z, Price KN, . Annual hazard rates of recurrence for breast cancer during 24 years of follow-up: results from the International Breast Cancer Study Group trials I to V. J Clin Oncol. 2016;34(9):927-935. doi:10.1200/JCO.2015.62.3504 26786933 PMC4933127

[4] Pan H, Gray R, Braybrooke J, ; EBCTCG. 20-Year risks of breast-cancer recurrence after stopping endocrine therapy at 5 years. N Engl J Med. 2017;377(19):1836-1846. doi:10.1056/NEJMoa1701830 29117498 PMC5734609

[5] Carter CL, Allen C, Henson DE. Relation of tumor size, lymph node status, and survival in 24,740 breast cancer cases. Cancer. 1989;63(1):181-187. doi:10.1002/1097-0142(19890101)63:1<181::AID-CNCR2820630129>3.0.CO;2-H 2910416

[6] Arriagada R, Le MG, Dunant A, Tubiana M, Contesso G. Twenty-five years of follow-up in patients with operable breast carcinoma: correlation between clinicopathologic factors and the risk of death in each 5-year period. Cancer. 2006;106(4):743-750. doi:10.1002/cncr.21659 16411216

[7] Contesso G, Mouriesse H, Friedman S, Genin J, Sarrazin D, Rouesse J. The importance of histologic grade in long-term prognosis of breast cancer: a study of 1,010 patients, uniformly treated at the Institut Gustave-Roussy. J Clin Oncol. 1987;5(9):1378-1386. doi:10.1200/JCO.1987.5.9.1378 3625256

[8] Frkovic-Grazio S, Bracko M. Long term prognostic value of Nottingham histological grade and its components in early (pT1N0M0) breast carcinoma. J Clin Pathol. 2002;55(2):88-92. doi:10.1136/jcp.55.2.88 11865000 PMC1769590

[9] Warwick J, Tabar L, Vitak B, Duffy SW. Time-dependent effects on survival in breast carcinoma: results of 20 years of follow-up from the Swedish Two-County Study. Cancer. 2004;100(7):1331-1336. doi:10.1002/cncr.20140 15042664

[10] Stuart-Harris R, Caldas C, Pinder SE, Pharoah P. Proliferation markers and survival in early breast cancer: a systematic review and meta-analysis of 85 studies in 32,825 patients. Breast. 2008;17(4):323-334. doi:10.1016/j.breast.2008.02.002 18455396

[11] Colozza M, Azambuja E, Cardoso F, Sotiriou C, Larsimont D, Piccart MJ. Proliferative markers as prognostic and predictive tools in early breast cancer: where are we now? Ann Oncol. 2005;16(11):1723-1739. doi:10.1093/annonc/mdi352 15980158

[12] van Diest PJ, van der Wall E, Baak JPA. Prognostic value of proliferation in invasive breast cancer: a review. J Clin Pathol. 2004;57(7):675-681. doi:10.1136/jcp.2003.010777 15220356 PMC1770351

[13] Bago-Horvath Z, Rudas M, Dubsky P, ; Austrian Breast and Colorectal Cancer Study Group. Adjuvant sequencing of tamoxifen and anastrozole is superior to tamoxifen alone in postmenopausal women with low proliferating breast cancer. Clin Cancer Res. 2011;17(24):7828-7834. doi:10.1158/1078-0432.CCR-11-1846 21998336

[14] Viale G, Giobbie-Hurder A, Regan MM, ; Breast International Group Trial 1-98. Prognostic and predictive value of centrally reviewed Ki-67 labeling index in postmenopausal women with endocrine-responsive breast cancer: results from Breast International Group Trial 1-98 comparing adjuvant tamoxifen with letrozole. J Clin Oncol. 2008;26(34):5569-5575. doi:10.1200/JCO.2008.17.0829 18981464 PMC2651094

[15] Jirstrom K, Ryden L, Anagnostaki L, . Pathology parameters and adjuvant tamoxifen response in a randomised premenopausal breast cancer trial. J Clin Pathol. 2005;58(11):1135-1142. doi:10.1136/jcp.2005.027185 16254100 PMC1770762

[16] Early Breast Cancer Trialists’ Collaborative Group. Tamoxifen for early breast cancer: an overview of the randomised trials. Lancet. 1998;351(9114):1451-1467. doi:10.1016/S0140-6736(97)11423-4 9605801

[17] Nordenskjöld A, Fohlin H, Fornander T, Lofdahl B, Skoog L, Stål O. Progesterone receptor positivity is a predictor of long-term benefit from adjuvant tamoxifen treatment of estrogen receptor positive breast cancer. Breast Cancer Res Treat. 2016;160(2):313-322. doi:10.1007/s10549-016-4007-5 27722840 PMC5065613

[18] Yu NY, Iftimi A, Yau C, . Assessment of long-term distant recurrence–free survival associated with tamoxifen therapy in postmenopausal patients with luminal A or luminal B breast cancer. JAMA Oncol. 2019;5(9):1304-1309. doi:10.1001/jamaoncol.2019.1856 31393518 PMC6692699

[19] Lindstrom LS, Yau C, Czene K, ; STO Trialists Group. Intratumor heterogeneity of the estrogen receptor and the long-term risk of fatal breast cancer. J Natl Cancer Inst. 2018;110(7):726-733. doi:10.1093/jnci/djx270 29361175 PMC6037086

[20] Esserman LJ, Yau C, Thompson CK, . Use of molecular tools to identify patients with indolent breast cancers with ultralow risk over 2 decades. JAMA Oncol. 2017;3(11):1503-1510. doi:10.1001/jamaoncol.2017.1261 28662222 PMC5710197

[21] Zhang XHF, Giuliano M, Trivedi MV, Schiff R, Osborne CK. Metastasis dormancy in estrogen receptor–positive breast cancer. Clin Cancer Res. 2013;19(23):6389-6397. doi:10.1158/1078-0432.CCR-13-0838 24298069 PMC3878717

[22] Fornander T, Rutqvist LE, Cedermark B, . Adjuvant tamoxifen in early breast cancer: occurrence of new primary cancers. Lancet. 1989;1(8630):117-120. doi:10.1016/S0140-6736(89)91141-0 2563046

[23] Rutqvist LE, Johansson H; Stockholm Breast Cancer Study Group. Long-term follow-up of the randomized Stockholm trial on adjuvant tamoxifen among postmenopausal patients with early stage breast cancer. Acta Oncol. 2007;46(2):133-145. doi:10.1080/02841860601034834 17453361

[24] Jerevall PL, Ma XJ, Li H, . Prognostic utility of HOXB13:IL17BR and molecular grade index in early-stage breast cancer patients from the Stockholm trial. Br J Cancer. 2011;104(11):1762-1769. doi:10.1038/bjc.2011.145 21559019 PMC3111159

[25] Barlow L, Westergren K, Holmberg L, Talback M. The completeness of the Swedish Cancer Register: a sample survey for year 1998. Acta Oncol. 2009;48(1):27-33. doi:10.1080/02841860802247664 18767000

[26] Emilsson L, Lindahl B, Koster M, Lambe M, Ludvigsson JF. Review of 103 Swedish healthcare quality registries. J Intern Med. 2015;277(1):94-136. doi:10.1111/joim.12303 25174800

[27] Swedish Breast Cancer Group. *National Guidelines for Treatment of Breast Cancer*. Version 3.0. Accessed December 14, 2020. http://www.swebcg.se/wp-content/uploads/2016/09/nationellt-vardprogram-brostcancer.pdf

[28] Hudis CA, Barlow WE, Costantino JP, . Proposal for standardized definitions for efficacy end points in adjuvant breast cancer trials: the STEEP system. J Clin Oncol. 2007;25(15):2127-2132. doi:10.1200/JCO.2006.10.3523 17513820

[29] Brenton JD, Carey LA, Ahmed AA, Caldas C. Molecular classification and molecular forecasting of breast cancer: ready for clinical application? J Clin Oncol. 2005;23(29):7350-7360. doi:10.1200/JCO.2005.03.3845 16145060

[30] Engelberg JA, Retallack H, Balassanian R, . “Score the Core” web-based pathologist training tool improves the accuracy of breast cancer IHC4 scoring. Hum Pathol. 2015;46(11):1694-1704. doi:10.1016/j.humpath.2015.07.008 26410019

