# Supplementary material for: Assessment of 25-Year Survival of Women With Estrogen Receptor–Positive/ERBB2-Negative Breast Cancer Treated With and Without Tamoxifen Therapy: A Secondary Analysis of Data From the Stockholm Tamoxifen Randomized Clinical Trial
Source: JAMA Netw Open. 2021 Jun 30;4(6):e2114904. doi: 10.1001/jamanetworkopen.2021.14904 (PMC8246315; doi:10.1001/jamanetworkopen.2021.14904)
Supplement: Supplement 1. — eMethods. Stockholm Tamoxifen (STO-3) Clinical Trial and Estrogen Receptor, Progesterone Receptor, ERBB2, and Ki-67 Immunohistochemistry eTable 1. Patient and Breast Cancer Tumor Characteristics by Treatment Arm eTable 2. Breast Cancer–Specific Survival by Clinically Used Breast Cancer Markers eFigure 1. Kaplan-Meier Analysis of Breast Cancer–Specific Survival eFigure 2. Breast Cancer–Specific Survival by Treatment Arm eFigure 3. Recursive Partitioning Survival Tree of Breast Cancer–Specific Survival [file jamanetwopen-e2114904-s001.pdf]

Supplementary Online Content

Dar H, Johansson A, Nordenskjold A, et al. Assessment of 25-year survival of women with estrogen receptor–positive/ERBB2-negative breast cancer treated with and without tamoxifen therapy: a secondary analysis of data from the Stockholm tamoxifen randomized clinical trial. *JAMA Netw Open*. 2021;4(6):e2114904. doi:10.1001/jamanetworkopen.2021.14904

eMethods. Stockholm Tamoxifen (STO-3) Clinical Trial and Estrogen Receptor, Progesterone Receptor, *ERBB2*, and Ki-67 Immunohistochemistry

eTable 1. Patient and Breast Cancer Tumor Characteristics by Treatment Arm

eTable 2. Breast Cancer–Specific Survival by Clinically Used Breast Cancer Markers

eFigure 1. Kaplan-Meier Analysis of Breast Cancer–Specific Survival

eFigure 2. Breast Cancer–Specific Survival by Treatment Arm

eFigure 3. Recursive Partitioning Survival Tree of Breast Cancer–Specific Survival

This supplementary material has been provided by the authors to give readers additional information about their work.

© 2021 Dar H et al. *JAMA Network Open*.

eMethods. Stockholm Tamoxifen (STO-3) Clinical Trial and Estrogen Receptor, Progesterone Receptor, *ERBB2*, and Ki-67 Immunohistochemistry

The Stockholm Tamoxifen (STO-3) trial

In the Stockholm Tamoxifen (STO-3) trial patients were randomized to receive adjuvant tamoxifen (40mg daily) versus no adjuvant treatment. In 1983, tamoxifen treated patients who reconsented and were recurrence-free after 2 years of tamoxifen therapy were randomized to 3 additional years of tamoxifen or no further therapy.

The patient subset with FFPE material available was well balanced to the original STO-3 trial cohort with regards to tumor characteristics, such as tumor size less than or equal to 20mm (78% vs 81%), ER-positive status (78% vs 80%), and tamoxifen-treatment arm assignment (52% vs 50%).[1]

ER, PR, ERBB2, and Ki-67 immunohistochemistry

Whole-tumor sections (5 micrometers) from FFPE tumor blocks were annotated in a random order at a single medical laboratory (University of California Davis Medical Center, UCDMC). The slides were stained using DAKO Link48 Autostainer and antibodies used were ER (SP1; Spring Bioscience M301), PR (PgR 636; DAKO IR068), ERBB2 (HercepTest; DAKO SK001), and Ki-67 (MIB-1; DAKO M7240), following standard recommended procedures including per-run positive controls.

eReference

1. Jerevall PL, Ma XJ, Li H, et al. Prognostic utility of HOXB13:IL17BR and molecular grade index in early-stage breast cancer patients from the Stockholm trial. *Br J Cancer*. May 24 2011;104(11):1762-1769. doi:10.1038/bjc.2011.145

© 2021 Dar H et al. *JAMA Network Open*.

eTable 1. Patient and Breast Cancer Tumor Characteristics by Treatment Arm

STO-3 Trial					
**Patients and tumor characteristics**	**Tamoxifen treated**		**Untreated**		**Association test**
	**Number**	**Percent**	**Number**	**Percent**	**Fisher's exact P-value**
**Calendar period of primary diagnosis**					0.50
**1976 - 1984**	150	50.3	142	53.2	
**1985 - 1990**	148	49.7	125	46.8	
**Age at primary diagnosis, year**					0.48
**45-54**	24	8.1	29	10.9	
**55-64**	152	51.0	136	50.9	
**65-74**	122	40.9	102	38.2	
**Tumor size**					0.41
**T1a/b**	84	28.5	84	31.8	
**T1c**	162	54.9	130	49.3	
**T2**	49	16.6	50	18.9	
**Unknown**	3	-	3	-	
**Tumor grade**					0.58
**1**	62	21.2	66	25.0	
**2**	194	66.2	167	63.3	
**3**	37	12.6	31	11.7	
**Unknown**	5	-	3	-	
**PR status[a]**					0.93
**Positive**	207	70.2	184	69.7	
**Negative**	88	29.8	80	30.3	
**Unknown**	3	-	3	-	
**Ki-67 status[b]**					0.59
**Low**	224	80.9	203	78.7	
**Medium/ High**	53	19.1	55	21.3	
**Unknown**	21	-	9	-	

[a]PR = Progesterone receptor status.
[b]Ki-67 threshold for medium/ high expression was 15% or greater.

© 2021 Dar H et al. *JAMA Network Open*.

eTable 2. Breast Cancer–Specific Survival by Clinically Used Breast Cancer Markers

25-year breast cancer specific survival (BCSS) by tumor size, tumor grade, PR status, and Ki-67 status, in patients with lymph node-negative and ER-positive/ ERBB2-negative breast cancer.

**Adjusted estimates for patient and tumor characteristics**				
**STO-3 trial**				
**Clinically used markers**		**Patients**	**Fatal breast cancer 25-year**	**Risk of fatal breast cancer (BCSS) HR (95% CI)[a,b]**
		**No.**	**No.**	
**Tumor size*[b]***	**$T1_{a/b}$**	168	15	**0.28 (0.15-0.54)**
	**$T1_c$**	292	58	**0.61 (0.39-0.96)**
	**T2**	99	30	1.0 ref.
**Tumor grade*[b]***	**Grade 1**	128	14	**0.38 (0.18-0.80)**
	**Grade 2**	361	66	0.60 (0.36-1.02)
	**Grade 3**	68	21	1.0 ref.
**PR status*[a,b]***	**Positive**	391	66	0.77 (0.51-1.16)
	**Negative**	168	37	1.0 ref.
**Ki-67 status*[a,b]***	**Low**	427	75	0.84 (0.52-1.38)
	**Medium/ High**	108	26	1.0 ref.

[a]HR = hazard ratio, CI = confidence interval, PR-positivity was defined as ≥10%, and Ki-67 threshold for medium/ high expression was 15% or greater.

[b]Modeled by multivariable Cox proportional hazard analysis adjusting for age at primary diagnosis, calendar period of diagnosis, tumor size, tumor grade, progesterone receptor (PR) status, Ki-67 status, and STO-3 trial arm.

© 2021 Dar H et al. *JAMA Network Open*.

eFigure 1. Kaplan-Meier Analysis of Breast Cancer–Specific Survival Kaplan-Meier analysis of breast cancer specific survival (BCSS) in patients with lymph node-negative and ER-positive/ ERBB2-negative breast cancer by; (A) Tumor size, (B) Tumor grade, (C) PR status, and (D) Ki-67 status. The P-value is based on a two-sided Log-rank test. Numbers at risk are shown underneath each graph.

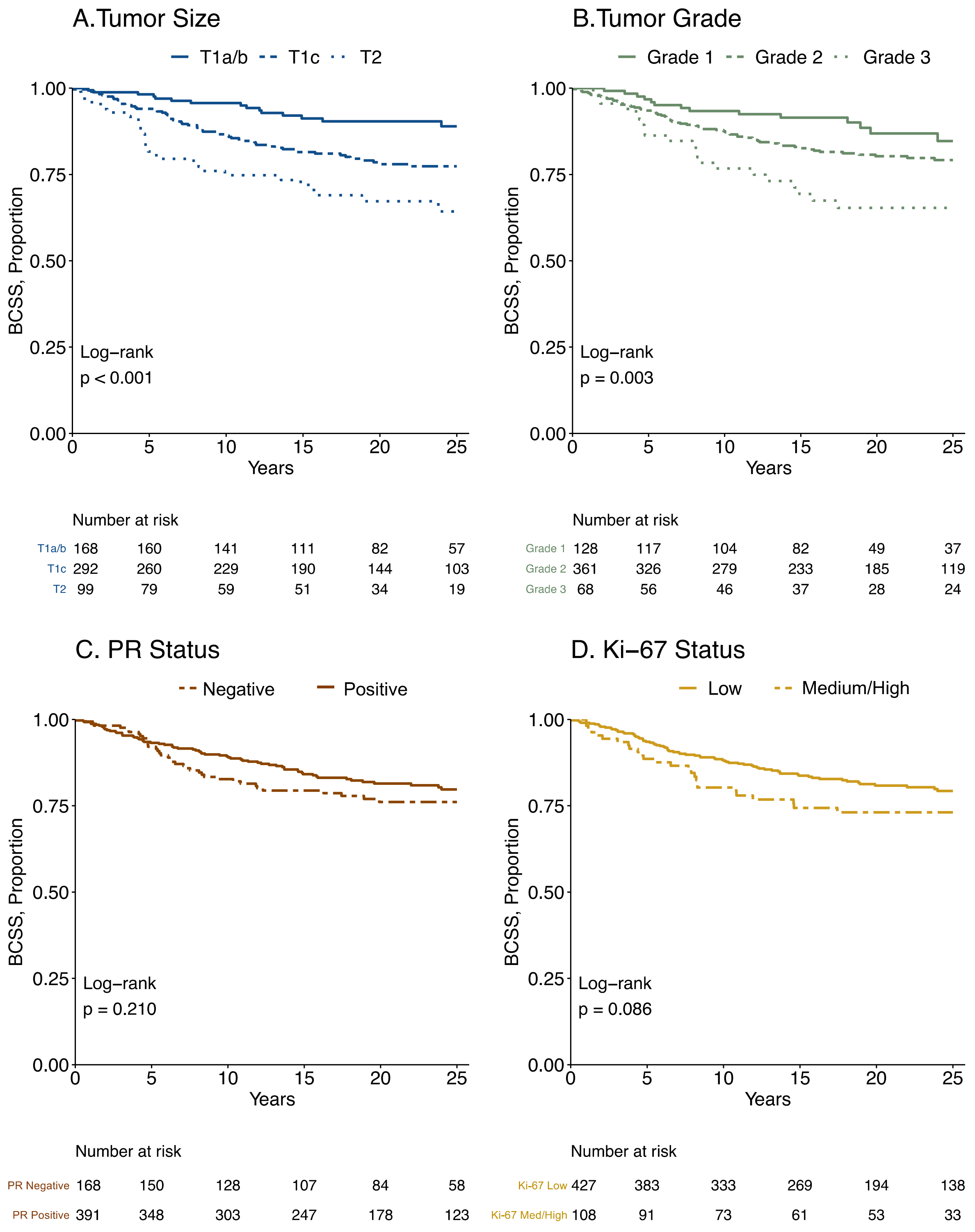

© 2021 Dar H et al. *JAMA Network Open*.

eFigure 2. Breast Cancer–Specific Survival by Treatment Arm 25-year breast cancer specific survival (BCSS) by STO-3 trial arm for tumor size, tumor grade, PR status, and Ki-67 status, in patients with lymph node-negative and ER-positive/ ERBB2-negative breast cancer.

Adjusted estimates for patient and tumor characteristics

**STO−3 trial** **Clinically used**	**markers**	**STO−3 trial arm**	**Patients No.**	**Fatal breast cancer 25 year No.**	**Risk of fatal breast cancer HR (95% CI)**
Tumor size	T1a/b	TAM treated	84	4	0.35 (0.11−1.17)
		Untreated	84	11	1.0 ref.
	T1c	TAM treated	162	25	0.54 (0.32−0.91)
		Untreated	130	33	1.0 ref.
	T2	TAM treated	49	10	0.41 (0.18−0.92)
		Untreated	50	20	1.0 ref.
Tumor grade	Grade 1	TAM treated	62	3	0.25 (0.06−1.08)
		Untreated	66	11	1.0 ref.
	Grade 2	TAM treated	194	26	0.53 (0.32−0.87)
		Untreated	167	40	1.0 ref.
	Grade 3	TAM treated	37	10	0.53 (0.21−1.33)
		Untreated	31	11	1.0 ref.
PR status	Positive	TAM treated	207	23	0.45 (0.27−0.74)
		Untreated	184	43	1.0 ref.
	Negative	TAM treated	88	16	0.54 (0.27−1.09)
		Untreated	80	21	1.0 ref.
Ki−67 status	Medium/High	TAM treated	53	7	0.28 (0.11−0.70)
		Untreated	55	19	1.0 ref.
	Low	TAM treated	224	30	0.55 (0.35−0.89)
		Untreated	203	45	1.0 ref.

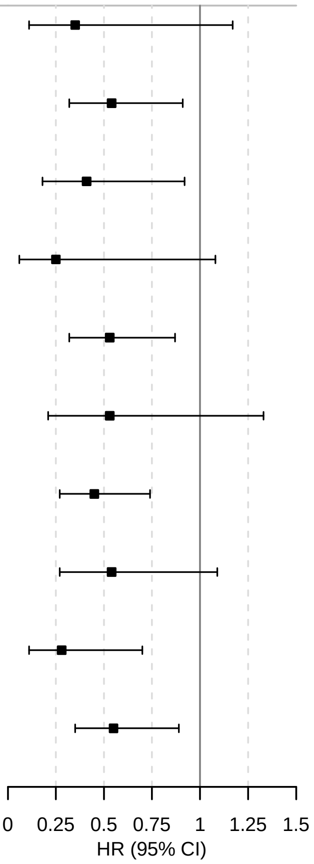

© 2021 Dar H et al. *JAMA Network Open*.

eFigure 3. Recursive Partitioning Survival Tree of Breast Cancer–Specific Survival

Recursive partitioning survival tree of breast cancer specific survival (BCSS) for patients with lymph node-negative and ER-positive/ ERBB2-negative breast cancer with associated Kaplan-Meier analysis.

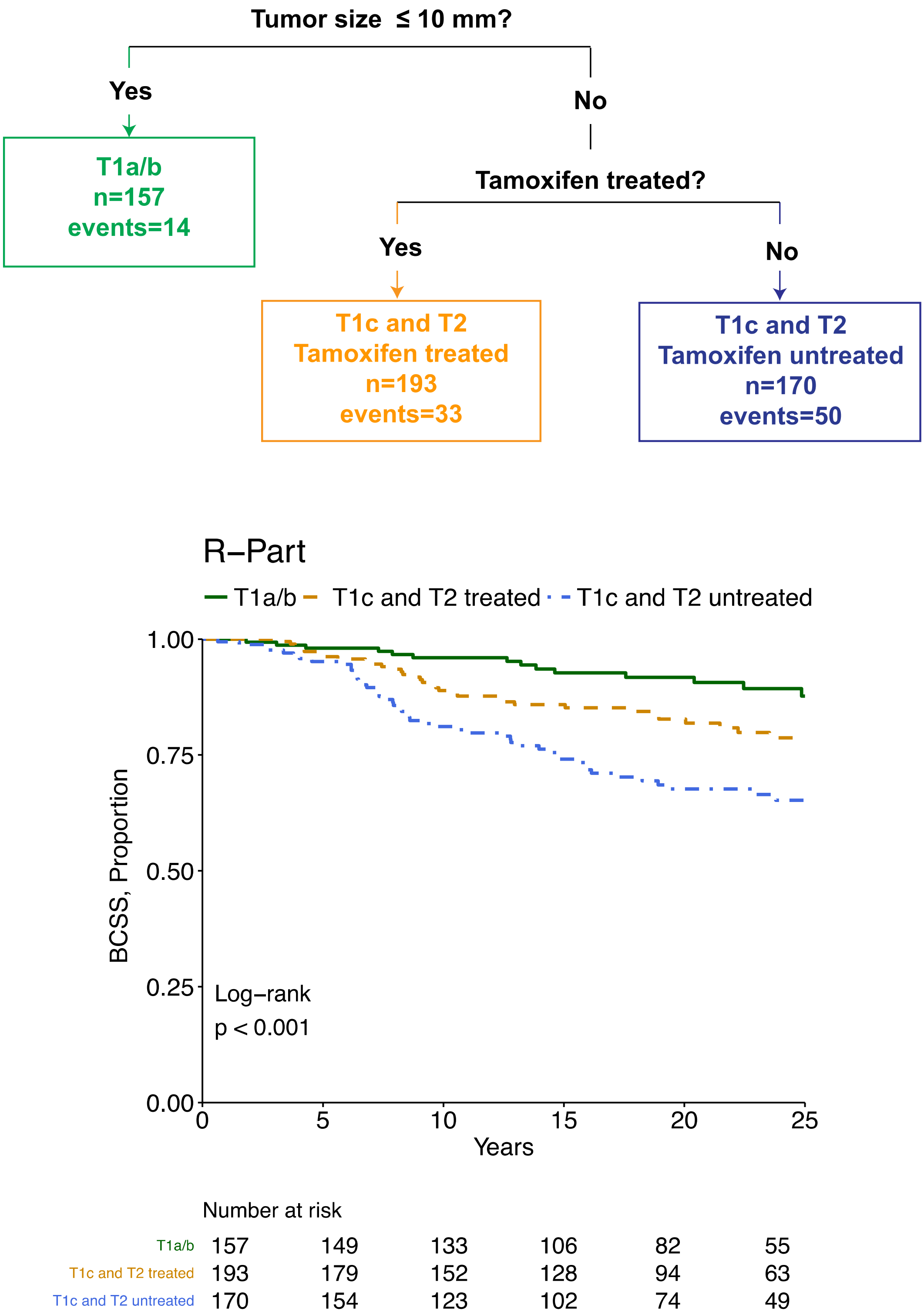

© 2021 Dar H et al. *JAMA Network Open*.
