# Supplementary material for: Assessment of 25-Year Survival of Women With Estrogen Receptor–Positive/ERBB2-Negative Breast Cancer Treated With and Without Tamoxifen Therapy: A Secondary Analysis of Data From the Stockholm Tamoxifen Randomized Clinical Trial
Source: JAMA Netw Open. 2021 Jun 30;4(6):e2114904. doi: 10.1001/jamanetworkopen.2021.14904 (PMC8246315; doi:10.1001/jamanetworkopen.2021.14904)
Supplement: Supplement 2. — Data Sharing Statement [file jamanetwopen-e2114904-s002.pdf]

Data Sharing Statement

Dar. Assessment of 25-Year Survival of Women With Estrogen Receptor-Positive/ERBB2-Negative Breast Cancer Treated With and Without Tamoxifen Therapy. *JAMA Netw Open*. Published June 30, 2021. doi:10.1001/jamanetworkopen.2021.14904

Data
Data available: No

Additional Information
Explanation for why data not available: Not possible due to GDPR within the EU.
